# Communities of practice in life sciences and the need for brokering

**DOI:** 10.12688/f1000research.7695.1

**Published:** 2016-03-04

**Authors:** Anne Tierney

**Affiliations:** 1 Department of Learning and Teaching, Edinburgh Napier University, Edinburgh, UK

**Keywords:** Community of Practice, brokering, collaboration, teaching and learning, pedagogy

## Abstract

Etienne Wenger’s work on communities of practice is influential in teaching and learning in higher education. A core work of many postgraduate certificate in teaching and learning (PGCert) courses for new lecturers, it is studied, in the main, as a means to understand how to support and encourage students to achieve more effective learning. Communities of practice can also be applied to academics. In the context of the Research Excellence Framework (REF) and its predecessors, the gulf between research-focused and teaching-Focused academics in life sciences has widened, so that in many institutions, these two groups have evolved into two distinct communities of practice; one whose priority is disciplinary research, the other’s learning and teaching. However, in 2015, the UK government announced that a Teaching Excellence Framework (TEF) would be introduced into higher education in England, as early as 2017. While the exact details of TEF remain unclear, it is certain that “excellence” and “student satisfaction” will be high on the agenda. It is vital, therefore, that the two communities of practice, research-focused and teaching-focused, find ways to come together in order to ensure high quality teaching and learning. Wenger proposes that this can be done through the process of “brokering”, which allows expertise from both communities of practice to cross from one to the other, strengthening both. This should be encouraged at departmental and institutional level, but another vital origin of brokering can be forged at a(n) (inter)national level at meetings such as the SEB Annual Conference, where teaching-focused academics have the opportunity to mix with research-active colleagues. While this paper is informed by recent and current events in the UK Higher Education sector, it is of interest to academics who work in an environment where research and teaching have become separate to any extent.

## Introduction

This paper is based on my experiences as a life sciences teaching-focused academic in a research intensive UK university, and my experiences working with organisations such as The Higher Education Academy, Quality Assurance Agency (Scotland) and The Society for Experimental Biology’s SEB+. It is also based on my observations of colleagues, and is related to my current doctoral work, although this paper represents a more general form of themes which have arisen from the study. The opinions contained in the paper are my own, and are influenced by the work of Etienne Wenger and Torgny Roxå and his colleagues. As such, my opinions and interpretations are open to discussion, and I welcome others’ thoughts on this subject.

## Communities of practice: focus on academics

Etienne Wenger’s work on communities of practice (1998) is influential in teaching and learning in higher education. It is used, primarily, to support student learning communities of practice, promoting the idea that learning is a social endeavour ( Bandura, 1977; Engeström, 1987; Parsons, 1962; Vygotsky, 1934). However, Wenger’s work can also be used to investigate how academics form communities of practice, the dimensions of which are defined by (1) mutual engagement; (2) joint enterprise; and (3) a shared repertoire ( Wenger, 1998, p. 73). In the UK, a number of external pressures, such as the Research Excellence Framework ( Higher Education Funding Council for England) and National Student Survey has increasingly meant the separation of research-focused and teaching-focused activities, resulting in the emergence of two distinct communities of practice; one whose enterprise priority is disciplinary research, the other, teaching and learning. The separation of joint enterprise coincides with differences in engagement and repertoire. Differences in engagement manifest as a withdrawal of research-focused academics from teaching, while teaching-focused academics reduce their involvement in, or are not required to engage in disciplinary research. Differences in repertoire can be seen as teaching-focused academics develop their expertise in the Scholarship of Teaching and Learning (SoTL), while research-focused academics develop their disciplinary expertise. Thus, the two groups diverge, forming two distinct communities of practice.

## Research-focused or teaching-focused: establishment of two communities of practice

Academics’ initial introduction to Wenger’s work is generally through the PGCert, which, post Dearing Report (1997) most institutions now require probationers to complete, although the absolute requirements for completion vary a great deal ( Gosling, 2010). At this point, individuals, although set on course to become members of either the research-focused or teaching-focused community of practice, are required to go through academic probation, which includes an introductory qualification in teaching and learning (PGCert). Completion of the PGCert, under its many guises, marks a departure point for academics; for many, it marks the end of a compulsory period of enquiry into pedagogy, and once the PGCert has been obtained, the business of establishing one’s disciplinary research reputation takes priority. For others, it marks the beginning of development of expertise in pedagogy, and the following of a teaching and scholarship career path. Thus, the two communities of practice diverge at this point. The divergence is aided by the pressures of the REF, which places emphasis on the production of world class disciplinary research ( Higher Education Funding Council for England), as well as differentiated career routes for staff (for example, the University of Glasgow has four academic career routes, designated “Research & Teaching”, “Research”, “Teaching & Scholarship” and “Veterinary Clinician”, all of which go as far as Professor (‘ Human Resources, College of MVLS’)).

While the separation of academics into research-focused and teaching-focused has its advantages, for example, allowing research-focused academics to concentrate on disciplinary research, while teaching-focused academics concentrate on teaching and learning, there are also disadvantages, research-focused academics do not develop their expertise in teaching and learning beyond the basics they are introduced to during their probationary period, and, while research-focused academics are still required to teach, this happens, in the main, in the form of didactic lectures. Teaching-focused academics, on the other hand, are required to develop their expertise in pedagogy, and are more inclined to introduce a variety of student-centred, active learning activities into their teaching, which supports student learning. However, this comes at the cost of developing their disciplinary expertise, as they leave active disciplinary research for pedagogy. Therefore there is a danger which impacts on the “student experience” – Research-focused academics whose disciplinary expertise is world class, but whose pedagogic knowledge is basic, and teaching-focused academics whose pedagogic expertise has been developed, but whose disciplinary expertise is hampered by no longer being research-active. While this has minimal impact on early years undergraduate education, it may impact on later years, in particular with undergraduate research projects, or with Masters level research projects. This situation has perpetuated over a number of years. However, there is a new influence coming to the higher education landscape, of England in particular, which may affect the two communities of practice, and, depending on institutions’ responses to it, has the potential to further divide the communities of practice, or act as a catalyst to unite them. This new influence is the Teaching Excellence Framework ( Johnson, 2015) which is proposed to be imposed on higher education in England as early as 2017. Despite education being a devolved matter, the influence of TEF is likely to be felt within institutions in all four home nations. While the details of the implementation of TEF remain unknown, the Green Paper sets great store in “excellence” and “student experience”, with one proposal being the introduction of an “Office for Students” ( Johnson, 2015, p. 62), to supersede The Quality Assurance Agency for Higher Education (QAA), one of its powers being “[to ensure] *the rights of students to hold providers to account and get value for money for their investment, and to protect them in the event of a provider exiting the sector*”. It is to be anticipated, therefore, that institutions will be subject to market forces, and students will be encouraged to seek “satisfaction”. Therefore, it is necessary to somehow realign the joint ventures of research-focused and teaching-focused academics in order to provide a research-led experience for students.

## Brokering: making connections across the boundaries

Wenger’s (1998) communities of practice do not operate in isolation. In reality, while communities of practice are distinct and can be identified as such, they work alongside one another, and mutually benefit from connections which are made across the boundaries between them. Connections between communities of practice are made in two ways, either via *boundary objects* or by *brokering*. Boundary objects are defined as “artifacts, documents, terms, concepts and other forms of reification around which communities of practice can organize their interconnections”, while brokering is defined as “connections provided by people who can introduce elements of one practice into another” ( Wenger, 1998, p. 105). As it stands, the research-focused and teaching-focused communities of practice share many boundary objects; a common knowledge base, common vocabulary and discourse, common or similar environments, equipment, methodologies. All of these serve as connections between the research-focused and teaching-focused communities of practice, identifying the participants as life scientists, albeit with a different primary focus of activities. In contrast, the opportunities for brokering remain limited, and the individuals who participate in the communities of practice remain, by and large, separated by the structures of the institution. Given that there are many boundary objects which the two communities of practice share, opportunities for brokering could be better facilitated. Opportunities to improve brokering necessitate the recognition of two distinct boundary objects which can be used as capital to facilitate exchange: The Scholarship of Teaching and Learning (SoTL), owned by the teaching-focused community and up-to-date Disciplinary Research, owned by the research-focused Community.

## Advantages of fostering brokering relationships between communities

Brokering relationships between teaching-focused academics and research-focused academics works to the advantage of both communities. While it would be naïve to claim that these relationships do not presently exist, it is my experience that they are neither common, nor long-lasting, but rather exist when convenient, on an ad hoc basis, for example, when a particular project or initiative is launched. However, given the proposals of the TEF to foreground teaching and learning and redress the imbalance between it and research, it becomes necessary to facilitate these brokering relationships in a more sustainable fashion. Thus, research-focused academics bring with them their latest research findings with which to inform an undergraduate learning activity, while teaching-focused academics bring the pedagogic underpinnings with which to make the learning experience valuable to the students. Simultaneously, as each brings their own expertise to the relationship, they also learn from one another, working at the periphery of each other’s expertise. This can work at the level of individual academics, working within a department, and at any level; postgraduate, postdoctoral fellow, probationer, lecturer, professor. Teaching-focused academics benefit from this arrangement by working with colleagues who have current research knowledge, while research-focused academics learn techniques which they can use to improve their teaching. The benefits go beyond the immediate exchange of expertise; graduate students who participated in undergraduate teaching saw improvements in their research methodological skills ( Feldon *et al.*, 2011). It may also be possible to extend this exchange by accommodating teaching-focused academics in research labs where they could update their research skills, improving their currency for subsequent years.

## Seeking excellence in teaching and learning

Given the competing pressures that academics work under, it is understandable why individuals may be sceptical of yet more work. However, the work of Roxå and colleagues ( Roxå & Mårtensson, 2011; Roxå *et al.*, 2007) suggests that there are two strategies which departments employ when attempting to improve “excellence” in teaching and learning, one of which is more likely to be more successful than the other. In what they term “Trajectory 1”, there are a few experts in pedagogy within a department who are relied upon by the colleagues to implement teaching and learning innovations. However, this leads to patchy application of pedagogic principles, which translates into differences in student experience. Roxå and his colleagues suggest that “Trajectory 2” represents a better strategy for departmental excellence in teaching and learning, by having every academic participate in it to a greater or lesser extent, thereby affecting departmental culture. In order for this to happen, it is necessary to broker relationships between Teaching-focused academics, who already have pedagogic knowledge, and research-focused academics, who have disciplinary research knowledge.

## The role of learned societies

Brokering also has the potential to work at a broader level, between institutions. In order for brokering relationships to be facilitated and encouraged, it is vital to have individuals from both communities of practice mixing together, and communicating their expertise. For life scientists, meetings such as those organised by learned societies are vital as a means for research-focused and teaching-focused academics to mix. For example, the SEB+ session at the 2015 SEB Annual Conference in Prague was a vibrant, well-attended day of presentations and discussion, notable for the mix of research-focused and teaching-focused academics in attendance. Plans are already in place for another SEB+ session in Brighton in 2016, based on its success in Prague. The SEB+ session offers a space where the issues of teaching and learning in a scholarly manner can be discussed in a supportive environment, and where good practice can be shared. While it is possibly too early to draw any conclusions from the session, if it gives research-focused academics an opportunity to reflect on their teaching practice, and sparks some level of interest in pedagogy, then it is to be encouraged, and accepted as a regular part of the conference programme.

## Conclusions

Disciplinary research has enjoyed a prioritisation within UK Higher Education, fuelled by the pressures of the REF. While the proposed TEF seeks to address this imbalance, and restore the status of teaching and learning in higher education, at this time it does not propose how it will do it. This paper has laid out a proposal, underpinned by pedagogic research theory, which suggests that closer working relationships between teaching-focused and research-focused academics is necessary in order to address “excellence”, by developing the disciplinary and pedagogic expertise of academics by facilitating the exchange of knowledge via expert relationships, which Wenger names “brokering”. The opportunities for brokering may take place within a department, but there is also a role for learned societies, such as The Society for Experimental Biology, to support brokering relationships between disciplinary and pedagogic practitioners.

I welcome discussion on this topic.
