# Supplementary material for: Communities of practice in life sciences and the need for brokering
Source: F1000Res. 2016 Mar 4;5:280. [Version 1] doi: 10.12688/f1000research.7695.1 (PMC4792202; doi:10.12688/f1000research.7695.1)
Supplement: Peer review history

# Peer review: Referee response for version 1

**DOI:** 10.5256/f1000research.8287.r12752

**Published:** 2016-03-22

**Authors:** Joy Perkins

**Affiliations:** 1 University of Aberdeen, Aberdeen, UK

This opinion piece is an extremely timely, topical and well-written article which highlights a range of important points regarding the two communities of practice (research-focused and teaching focused) which exist within universities and across the HE sector. There clearly needs to be a re-balance between the tensions of teaching and research and the author proposes approaches to help meet and unite the needs of both communities of practice through for example, an enhanced role for learned societies. To strengthen the article a paragraph recognising the role of the UK Professional Standards Framework (UKPSF) and the Higher Education Academy (HEA) Fellowship recognition scheme in raising the profile of teaching to all academics would be a valuable addition. HEA Fellowship status is a quality assurance indicator in UK universities regarding standards in teaching and supporting students’ learning and is often implemented as a KPI target in many institutions. Research experience is often relevant to an individual’s HEA Fellowship application if it relates to their university learning and teaching. Is this a further opportunity to extend the ‘brokering model’ proposed by the author? There is also potential to unite both communities of practice at HEA Writing Retreats and HEA recognition events, these CPD opportunities help support both communities to network and communicate their expertise.

The article is acceptable for approval with a few minor corrections, it should be the Society for Experimental Biology (lower case t) – Introduction section, line 3 and the same applies to the Higher Education Academy (Introduction section, line 2).

I have read this submission. I believe that I have an appropriate level of expertise to confirm that it is of an acceptable scientific standard.

# Response

**Published:** 2016-03-23

**Authors:** Anne Tierney

**Affiliations:** Edinburgh Napier University, UK

The suggestion to include UKPSF and HEA fellowship is a sensible one, and I shall include a paragraph in the paper. Thank you very much for the suggestion, and thank you for taking the time to review the paper.

# Peer review: Referee response for version 1

**DOI:** 10.5256/f1000research.8287.r12753

**Published:** 2016-03-17

**Authors:** Morven C. Shearer

**Affiliations:** 1 School of Medicine, University of St Andrews, St Andrews, UK

This is a thoughtful and timely piece, looking at the different communities of practice (CoPs) that exist in our HEIs currently and arguing that these communities must work together (despite the pressures of REF and, the potential-TEF, drawing them apart) to achieve excellence in teaching and learning. I think Tierney has raised good points, grounded in the literature around CoPs, concerning the need for 'brokering relationships' and highlighting the very real threat to HE without such action. Both types of practitioners are equally valued and respected in her discussion and drawing on theory, policy and experience this article forms a good opinion piece. It'll be interesting in particular to see what further study of the contribution and activity of learned societies in this area reveals.

I have read this submission. I believe that I have an appropriate level of expertise to confirm that it is of an acceptable scientific standard.

# Response

**Published:** 2016-03-23

**Authors:** Anne Tierney

**Affiliations:** Edinburgh Napier University, UK

The area of learned societies in one within which there is a lot of potential to support teaching and learning, and many learned societies include such in their conferences. We shall see over the coming years what the outcome of this is, as there is an appetite for support, which I believe will increase once the substance of TEF is known.

Thank you for the review, and I look forward to any comments you have on this in the future.

# Peer review: Referee response for version 1

**DOI:** 10.5256/f1000research.8287.r12751

**Published:** 2016-03-14

**Authors:** Kevan M.A. Gartland

**Affiliations:** 1 Life Sciences, Glasgow Caledonian University, Glasgow, UK

This Opinion Article raises a number of very interesting points about the inter-relationship between research academic specialists and those pursuing a learning and teaching led specialism. It highlights the tensions that may frequently emerge between the two and proposes the use of brokering as a way forward, in the context of the emergent Teaching Excellence Framework (TEF), for universities in England, which will also have impacts and change behaviours in the rest of the UK and further afield. The author emphasises the value of enhancing relationships between these two communities of practice, and the role that Learned Societies, for example, can play to promote greater interchange of ideas and experiences between these groups. Without such initiatives, these groups which may also extend beyond single departments and across whole institutions, may diverge further leading to a paucity of student learning experience and the detriment of lost knowledge, skills and expertise that might not meet institutional Research Excellence Framework priorities. The rise of the TEF provides an opportunity for the imbalance of priorities between research and learning & teaching which may arise, to be redressed for the benefit of all. The article explains the opinions stated well, placing them into the context of previous work. The conclusions are balanced and justified. The article is acceptable for approval, subject to the following minor corrections: 'teaching-focused' (Abstract, line 7 of pdf), 'community' (lower case 'c'; at end of para 1 page 4 of pdf), and 'teaching-focused' (para 3, page 4 of pdf).

I have read this submission. I believe that I have an appropriate level of expertise to confirm that it is of an acceptable scientific standard.

# Peer review: Referee response for version 1

**DOI:** 10.5256/f1000research.8287.r12754

**Published:** 2016-03-08

**Authors:** Peter Klappa

**Affiliations:** 1 School of Biosciences, University of Kent, Canterbury, UK

I think this is a very well written opinion article, which is underpinned by the relevant research in Higher Education, especially pertinent to the work of Eric Wenger *et al. *The concept of 'brokering' to bridge the gap between the different communities of practice is very important, especially in a time of tension between research and teaching. It will be very interesting to see, how this concept is transformed into reality through emerging examples and case studies.The article should be indexed as is without any further corrections.

I have read this submission. I believe that I have an appropriate level of expertise to confirm that it is of an acceptable scientific standard.
